# The history and status of dopamine cell therapies for Parkinson's disease

**DOI:** 10.1002/bies.202400118

**Published:** 2024-07-26

**Authors:** Roger A. Barker, Anders Björklund, Malin Parmar

**Affiliations:** ^1^ Department of Clinical Neurosciences and Cambridge Stem Cell Institute John van Geest Centre for Brain Repair University of Cambridge Cambridge UK; ^2^ Department of Experimental Medical Science Wallenberg Neuroscience Center Lund University Lund Sweden; ^3^ Department of Clinical Sciences Lund Lund Stem Cell Center and Division of Neurology Lund University Lund Sweden

**Keywords:** clinical trials, dopamine cell transplants, G Force PD, human fetal ventral mesencephalic tissue, human pluripotent stem cell‐derived dopamine cells, Parkinson's disease

## Abstract

Parkinson's disease (PD) is characterized by the loss of the dopaminergic nigrostriatal pathway which has led to the successful development of drug therapies that replace or stimulate this network pharmacologically. Although these drugs work well in the early stages of the disease, over time they produce side effects along with less consistent clinical benefits to the person with Parkinson's (PwP). As such there has been much interest in repairing this pathway using transplants of dopamine neurons. This work which began 50 years ago this September is still ongoing and has now moved to first in human trials using human pluripotent stem cell‐derived dopaminergic neurons. The results of these trials are eagerly awaited although proof of principle data has already come from trials using human fetal midbrain dopamine cell transplants. This data has shown that developing dopamine cells when transplanted in the brain of a PwP can survive long term with clinical benefits lasting decades and with restoration of normal dopaminergic innervation in the grafted striatum. In this article, we discuss the history of this field and how this has now led us to the recent stem cell trials for PwP.

## INTRODUCTION

Parkinson's disease (PD) is a chronic neurodegenerative disorder of the brain that takes its name from James Parkinson who first described it in 1817.^[^ ^1^ ^]^ It is characterized by the presence of motor abnormalities (rigidity, rest tremor, bradykinesia, and postural instability) although is now recognized to have a host of non‐motor features, some of which can precede the onset of the motor abnormalities by which it is diagnosed.^[^ ^2^ ^]^ These prodromal features (REM sleep behavioral disorder, constipation, hyposmia) have attracted much interest of late as they offer a window of opportunity for the trialing of potentially disease modifying therapies before the disease has taken a major hold on the CNS pathologically. However, to date, there are no confirmed early diagnostic nor disease modifying therapies for people with Parkinson's (PwP) and so they are managed symptomatically using agents that target the core pathology of PD, namely the loss of the dopamine neurons in the nigrostriatal pathway.

The adult human substantia nigra pars compacta (SNpc) contains between 400 000 and 500 000 dopamine neurons on each side^[^ ^3^ ^]^ and once half of these cells are lost and 80% of their projection to the putamen, then the patient presents with their early diagnostic motor features. Once diagnosed, patients are typically treated with a combination of dopaminergic drugs (L‐dopa, dopamine agonists, MAO, and COMT inhibitors), often with great success in the early stages of the disease.^[^ ^4^ ^]^ However, with time these drugs start to lose their efficacy as the loss of cells within the CNS progresses, including systems outside this dopaminergic pathway. In addition, these dopaminergic agents produce two major side effects. The first relates to neuropsychiatric problems due to the drugs stimulating intact dopaminergic networks (e.g., in the cortex and ventral striatum). The second is the generation of involuntary movements (e.g., L‐dopa induced dyskinesias – LIDs) due to the non‐physiological stimulation of the dopamine receptors by the orally taken drugs within the striatum (including the putamen). This in turn requires further interventions either orally using drugs such as amantadine to suppress the LIDs, parenteral dopaminergic infusion therapies (e.g., infusions of subcutaneous apomorphine or L‐dopa or intrajejunal DuoDopa), or neurosurgery (e.g., deep brain stimulation [DBS]).^[^ ^4^, ^5^ ^]^

This dopaminergic approach to treating PD, which began in the 1960s, still remains the mainstay in 2024, and although new advances are being made in the development of other therapies (e.g., adaptive DBS), there is an urgent need to find not only disease‐modifying therapies but better symptomatic and regenerative dopaminergic treatments. In this last respect, dopamine cell transplants have been an area of great interest given that they offer the potential for patients with Parkinson's (PwP) to have: (i) a one‐off therapy that should work as well as their oral dopamine therapies but (ii) without the risk of off‐target effects or LIDs, given that such grafts would be neuronal in nature and placed at the site of greatest dopamine loss in PD – namely the putamen. Furthermore, given the number of cells that would be needed to effect functional repair of this network, namely 100 000 to 200 000 dopamine (DA) neurons, then this approach has clear practical attractions. In this article, we will lay out the history of how dopaminergic cell repair of PD brain has evolved from preclinical studies starting in the mid‐1970s to a number of first‐in‐human trials of human stem cell‐derived dopaminergic neurons in 2024.

However, before this discussion, it is important to remember that this approach is not trying to cure PwP, as the underlying alpha‐synuclein pathology of PD is not being targeted. Nor is it trying to treat all the symptoms and signs of PD as these take their origin from pathologies across many CNS sites as well as outside the brain including in the enteric and autonomic nervous systems.^[^ ^6^ ^]^ What it is trying to do, is to repair the core pathological feature by grafting in new nigral dopamine cells of the type lost to the disease process and replacing their function. Since age is the single biggest risk factor for the development of PD^[^ ^7^ ^]^ it seems possible that their young age would make them less prone to be affected by the ongoing disease process. However, this assumption is only partially true as alpha‐synuclein pathology has been seen to develop after 10+ years post‐transplantation of human fetal midbrain tissue into the brain of PwP.^[^ ^8^, ^9^ ^]^ Ways to make the cells resistant to the development of α‐synuclein pathology have started to be addressed with the new generation of stem cell‐derived dopamine cells – see below.^[^ ^10^ ^]^

## THE EARLY HISTORY OF DOPAMINE CELL THERAPIES FOR PwP; 1970–2000

The early days of the dopaminergic cell therapy approach have been summarized previously^[^ ^11^ ^]^ and thus will not be dealt with in great detail here. Nevertheless, a summary of this early work is warranted as it frames much of what has happened in the 21st century around this therapeutic strategy.

The early pioneering work using transplants of fetal neurons for brain repair was undertaken by Anders Björklund and his team in Lund, Sweden in the 1970s.^[^ ^12^, ^13^ ^]^ The purpose of these early brain repair studies was to define the conditions for various types of neuroblasts, taken from the developing brain, to survive and establish functional connections after implantation into the adult brain. These studies were made possible around this time by the Falck‐Hillarp histofluorescence technique which allowed for the visualization of monoaminergic pathways – including the dopaminergic system – in their entirety. On this background the work began, although at the same time other work was starting to look at a range of other dopamine cell replacement therapies for PD, especially using adrenal medullary chromaffin cells. The rationale for this latter work was that these cells could produce dopamine, albeit at low concentrations, and could be grafted in an autologous fashion from the abdominal cavity into the striatum of PwP (reviewed in Ref. [14]). However, over time it became clear that using non neuronal cells to repair the brain, especially in PD, was sub‐optimal and that using fetal dopamine cells offered the best approach.

The pioneering preclinical work using fetal dopamine cells, performed in Björklund´s lab,^[^ ^15^, ^16^ ^]^ began in earnest in the early 1980s, showing that this dopamine cell replacement approach worked in the unilateral medial forebrain bundle 6‐OHDA lesion model of PD. This model, which had been developed by Urban Ungerstedt in the 1970s,^[^ ^17^ ^]^ works through the irreversible loss of the DA nigrostriatal projection with a simple functional readout using drug‐induced rotation tests. This model is not one of PD, but is a very useful one for studying the functionality of the nigrostriatal dopaminergic pathway which is what one is trying to repair using dopamine cell therapies. As such, it is an excellent model for studying this reparative approach to PD, and it still remains the mainstay for preclinical studies testing stem cell‐derived dopaminergic therapies (see e.g., Ref. [18, 19]).

This preclinical work showed that rat fetal ventral mesencephalic tissue, containing the developing nigral dopaminergic neurons, could survive when grafted into the dopamine denervated adult rat striatum. This survival was optimal when the tissue was allografted and taken at the time when the developing dopamine neurons were entering their last division before differentiating into dopaminergic neurons (reviewed in Ref. [20]). In addition, this survival was associated with synaptic innervation to (and from) the host and the release of dopamine with functional recovery as long as a critical number of cells survived with innervation of the surrounding striatum. This work was then extended to study human fetal ventral mesencephalic tissue (hfVM) in this same model system, and similar results were seen, with the main difference being that these cells took longer to develop and innervate, compared to the rodent equivalent cells – at least 24 weeks as opposed to 8 weeks for functional recovery on drug‐induced rotation.^[^ ^21^ ^]^ Furthermore, these human dopaminergic cells also showed much greater capacity to extend fibers as one would expect given the degree of innervation they need to achieve in the developing human brain. The capacity of human dopamine neurons – derived either from fetal VM tissue or pluripotent stem cells (PSCs) – to extend axons over large distances has elegantly been shown in experiments where these cells have been grafted into the rat or mouse nigra and then found to innervate the relevant cortical areas as well as the striatum of the transplanted rat several months later.^[^ ^22^, ^23^, ^24^, ^25^ ^]^ The value of this preclinical approach is clear as it is still being used to assess the new generation of human stem cell‐derived dopaminergic neurons often against that which is seen with contemporaneous hfVM tissue grafts.

The translational work, which required establishing a new ethical framework for working with human fetal tissue collected from termination of pregnancies in Sweden, led to clinical trials that began towards the end of the 1980s. This was undertaken in a number of centers around the world including Mexico, France, the USA, and Canada but with the team in Lund leading the way but with patients that also were recruited from, and then followed up in, Germany and the UK.^[^ ^26^ ^]^ Indeed, this latter collaboration between the UK and Sweden is one that has continued to the present day with the next generation of scientists and clinician scientists (Anders Björklund, Olle Lindvall, Stig Rehncrona, David Marsden, Paola Piccini, Wolfgang Oertel, and Niall Quinn, giving way to Malin Parmar, Agnete Kirkeby, Hakan Widner, Gesine Paul, Hjalmar Bjartmarz and Roger Barker).

The initial open label trials using this approach with hfVM tissue showed encouraging results, with evidence of graft survival and in some cases normalization of striatal dopamine levels, thus allowing patients to come off all their anti‐PD medication (reviewed in Ref. [20, 27]). However, it was also clear that the results were variable and some better optimized approach was needed for this variance to be reduced, if this was possible. In 1993, when Bill Clinton entered the White House, federal funding for the use of hfVM tissue was permitted in the USA, allowing NIH to fund two double blind randomized control trials investigating the safety and efficacy of hfVM as a therapeutic approach in PD. These trials published their results in 2001 and 2003^[^ ^28^, ^29^ ^]^ and reported that the therapy was ineffective in terms of achieving its primary end point and in addition produced significant side effects in terms of graft induced dyskinesias (GIDs) in a significant proportion of patients in both trials. As such, by 2003, interest in this therapeutic area was waning and at the same time the use of DBS was growing following a number of successful trials using this approach in patients with advancing PD (early review in Ref. [30, 31]). In addition, in 2008, reports appeared showing that the some of the dopamine cells within the hfVM grafts had acquired the alpha synuclein pathology of PD.^[^ ^32^, ^33^ ^]^ The reason for this was not clear, and is still not fully known, but was thought to relate to pathological alpha synuclein spreading from the host PD brain into the grafted cells, where it then templated pathology using the grafted cells own endogenous alpha synuclein. This prion like behavior for alpha synuclein has spurned a whole new field of research in PD (see Ref. [34]), but for our discussion here it is important to recognize that this is only seen after more than 10 years post implantation and even in the longest surviving graft analyzed at post mortem after 24 years, the vast majority of the transplanted dopaminergic cells did not display such pathology.^[^ ^8^ ^]^ Thus, although a fascinating observation, this pathology is not in itself a reason not to continue with this approach. At least not when using cells from healthy donors, given the slow speed with which this pathology appears in transplanted healthy dopaminergic neurons. Nevertheless for those critical of this whole therapeutic approach, this was another reason for not pursuing it and contributed to the negative perception of this field in the early part of this century.^[^ ^35^ ^]^

## THE PROBLEM YEARS 2000–2010

As stated above, the future of dopamine cell therapies for PD hung in the balance in the early part of this century and it was unclear whether this approach had any merit given the emergence of DBS. As part of this re‐appraisal of the field, Roger Barker and Anders Björklund decided to set up a working group in 2006 that sought to bring together all those who had been involved in hfVM transplant trials in PD over the previous 20 years with the aim of seeing whether this dopamine cell therapy approach had any merit. This group had its first meeting at the John van Geest Centre for Brain Repair in Cambridge in 2006 which was then followed by a series of similar workshops in London funded by Parkinson's UK. In these meetings, chaired by Roger Barker, representatives from the clinical trial teams based in Lund, Paris, and the USA attended as well as interested parties from within the UK and Michael J Fox Foundation. These meetings sought to critically re‐evaluate the data pertaining to all these hfVM trials and decide what had, and had not, been shown in these trials and whether dopamine cell therapies for PwP had any future. Similar individual meetings were also being organized by others including the Michael J Fox Foundation in 2007 in London and NIH in 2010 in Washington – where other aspects of trial design were discussed including the need for sham/imitation surgery.^[^ ^36^ ^]^

As a result of these discussions, a number of conclusions started to emerge. First, while DBS was effective, it is not regenerative nor is it without problems, thus indicating that other non‐DBS approaches had some merit. Second, some patients had had a significant long‐lasting benefit from the hfVM transplants, and this was associated with improved dopamine signal in the grafted striatum on PET imaging and a reduction or cessation of their anti‐PD medication (e.g., Ref. [26]). Thirdly, GIDs were a real concern, and understanding their genesis was important if the field was ever to be able to move forward with confidence. On balance, this was felt to be due to contaminating 5HT neurons in the transplant and the number of such cells relative to the number of surviving dopaminergic neurons.^[^ ^37^ ^]^ However, alternative theories for GIDs were also posited including the generation of dopamine hot spots at the transplant site within the grafted striatum.^[^ ^38^ ^]^ Fourthly, the variability between trials could be explained in part by the amount of tissue grafted, how the tissue was processed and stored, the level and duration of immunosuppression post‐transplantation, the length of follow‐up, and the type of patient recruited into the trial.^[^ ^27^ ^]^ Finally, it was recognized that the use of hfVM tissue to repair the PD brain was never going to become a standard of care for PwP, even if one could get more consistent results, because of ethical and logistical issues with acquiring sufficient amounts of this tissue in a standardized way. Nevertheless, all this work gave teams in Europe the confidence to apply for funding for a new trial in PwP using hfVM with the aim of demonstrating that attention to the above factors could give a more consistent response. The initial application to do this was turned down by the Michael J Fox Foundation on the grounds that “it was more of the same” but the EU agreed to fund this new work in a study called TransEuro that began in 2010.^[^ ^39^ ^]^

## TransEuro 2010–2024

This new EU‐funded project involved groups of clinicians and scientists from a number of UK and European sites – Cambridge, London (UCL and ICL), Cardiff, Lund, Paris, and Freiberg with an ethical input from Vienna. There were two main aims of TransEuro: (i) to undertake a natural history study of ∼150 PwP who were thought to be optimal for neural grafting with dopamine neurons. This group was identified from our previous epidemiological work in PD^[^ ^40^ ^]^ as well as our limited meta‐analysis of previous hfVM trials^[^ ^27^ ^]^; (ii) undertake two transplant trials using hfVM tissue. One would be an open‐label study of 20 patients within Europe followed by a second randomized control trial of 60 patients involving an independently funded arm in the US. The patients in the transplant studies would be drawn from the natural history cohort who were being followed up with an array of clinical assessments as well as functional MRI and PET imaging in a sub‐group of these patients. This project was funded for 5 years but the entire program is still ongoing 14 years since it started, having secured additional support most notably from the MRC for imaging and the Cure Parkinson charity for the follow‐up of the patients (both natural history and grafted individuals).

Ultimately TransEuro delivered on its natural history study^[^ ^39^ ^]^ but failed to complete the transplant trials as initially conceived. In fact, only the first open label trial was achieved but with 11 patients, instead of the planned 20. The first patient was grafted in 2015 in Cambridge and the last in 2018 in Lund. The results from this study are currently in revision for publication, but the reasons for the trial delay are summarized in Information Box 1. Most importantly for our discussion here, was the issue of tissue supply. Despite using tissue from both medical and surgical terminations of pregnancies from >1 center,^[^ ^41^ ^]^ the amount of tissue that could be secured (at least three hfVM over a 4 day period) to enable a transplant to proceed within the set time limit became a rate limiting step. Eighty‐seven surgeries were cancelled because of insufficient material being available for transplantation. No patients were grafted with four hfVMs per side, as this never became available within the trial. As it transpired, looking at the data post grafting, the choice of three hfVM proved to be too low a dose to achieve a significant repair in the majority of patients. This highlighted again the logistical problems of using hfVM tissue, such that it could never be relied upon for treating PwP. A point that was emphasized by the fact that we also saw that some patients developed GIDs despite trying to restrict our tissue dissection to minimize any contamination of the grafted tissue with significant numbers of the developmentally neighboring 5HT neurons. When working with stem cells (see below), 5HT neurons can be completely avoided in the cell transplant.

**Information Box 1: Complications causing delays in the TransEuro hfVM transplant trial**

**Regulatory**:

- Delay in getting a response from MHRA as to whether we were using an advanced therapy medicinal product (ATMP);
- Problems with different countries seeing the trial differently from a regulatory perspective;
- New documents/data unexpectedly required by the HTA for fetal tissue processing in the UK;
- Delays in getting renewal of permits for fetal tissue work in Sweden;
- Problems in the coordination across sites with tissue transfers esp. post Brexit.

**Management issues**:

- Change in status of the EU funded partners which necessitated getting a new partner for our PET imaging;
- Management company overseeing our EU project went into liquidation;
- A work package leader died;
- A PI left their position prematurely with a major budget deficit at that site linked to our project.

**Trial/tissue procedures**

- Delays in developing a new clean room in Sweden;
- Unexpected breakdowns in equipment in the GMP lab in the UK;
- Unexpected problems in reproducibility of tissue preps across sites – leading to additional studies needing to be done to rectify this problem;
- In the UK, a change in the hospital policy on insurance for the surgical cell delivery device – meant we had to make a new device in the UK based on one being used in Sweden;
- UK hospital moved to a new electronic system for all patients (EPIC) and during this time all elective surgery was cancelled for many months;
- Problem with sterilization of device by the UK hospital, meaning some devices were not ready for when surgery was booked;
- Neurosurgical theatre refurbishment in the UK meant all neurosurgery had to vie for space in hospital main theatres;
- Junior doctors strike in the UK stopped all elective and research surgery/studies;
- Problems with reagents passing their validation date because of delays from above;
- Tissue availability was a major issue and we ultimately undertook 21 surgeries in cases where we had enough tissue but another 87 booked surgical sessions were cancelled because of insufficient tissue.

**Problems with patient follow‐up**

- The COVID pandemic led to the cancellation of many follow‐up appointments and PET scans.

Thus TransEuro, whilst telling us a lot about how to better design and execute trials delivering dopamine cells into the brains of patients with PD, failed to show efficacy and also proved that using hfVM was never going to be a viable tissue source of dopamine neurons for PD repair. The question then arises as to why we chose to do this trial rather than use hPSC derived dopamine cells. The answer is that when TransEuro was funded in 2009, no protocols existed for generating such cells from a human PSC source – although this all changed in 2011–2012.

## THE BREAKTHROUGH IN hPSC DOPAMINE CELL DERIVATION 2011‐

PSCs have two distinguishable characteristics, self‐renewability, and pluripotency, allowing them to (theoretically) give rise to almost any cell type in the adult body. There are two major types of human PSCs, human embryonic stem cells (hESCs^[^ ^42^ ^]^) and human induced PSCs (hiPSCs^[^ ^43^ ^]^) both of which are currently being explored for cell transplantation in PwP.

The midbrain DA neurons are born during a narrow window of early brain development (E10.5–12 in the mouse; 6–8.5 weeks p.c. in humans). Initial protocols for generating DA neurons from human PSCs were based on adapting mouse ES differentiation protocols. This included induction of neural differentiation via embryoid body (EB) formation^[^ ^44^ ^]^ the use of feeder cells^[^ ^45^ ^]^ and timed delivery of morphogens and growth factors, for example, SHH, FGF8, BDNF, and GDNF^[^ ^46^, ^47^ ^]^ to induce DA neurons via a PAX6 positive intermediate. While these protocols gave rise to DA neurons in vitro, their midbrain identity was not confirmed and the cells performed poorly after transplantation.^[^ ^47^, ^48^, ^49^ ^]^ With the insight that midbrain DA neurons actually develop from floorplate progenitors that co‐express FOXA2/LMX1A and not from neuroepithelial cells expressing PAX6,^[^ ^50^, ^51^, ^52^ ^]^ new protocols for the generation of midbrain DA neurons were developed. These were based on dual SMAD inhibition for neuralization and early exposure to SHH for ventralization and floor plate formation.^[^ ^53^ ^]^ When combined with activation of the WNT pathway and Fgf8 for caudalization, it was possible to produce midbrain patterned DA neurons that survived, regrew the nigrostriatal pathway, and reversed dopamine‐related motor deficits in animal models of PD after transplantation.^[^ ^23^, ^54^, ^55^ ^]^ A number of subsequent efforts have been devoted to optimizing DA differentiation by modifying the culture conditions, for example by using different concentrations and timing of CHIR99021 (CHIR), introducing Fgf8, and/or introducing cell sorting.^[^ ^56^, ^57^, ^58^, ^59^, ^60^, ^61^, ^62^ ^]^

## G FORCE PD 2014‐

In order to avoid some of the problems that arose in the development of hfVM derived transplants for PwP, in 2014 a new forum was set up that brought together the major academic groups working on taking human pluripotent stem cell (hPSC) derived dopamine cells to first in human clinical trials. The main groups that met for the first time in London in 2014, were the teams of Malin Parmar from Lund, Sweden funded through an EU grant called NeuroStemCellRepair; Roger Barker and the TransEuro team from Cambridge and Imperial College (for the PET imaging); Lorenz Studer and Viviane Tabar who were funded by NYSTEM at that time, and Jun Takahashi and colleagues from CIRA in Kyoto in Japan who were funded by their institution. These meetings have been held annually ever since in different locations with the aim to better inform the teams on progress and problems in translating their hPSC dopamine cell product to clinic. This consortia have produced joint publications highlighting how a first in human trial with these cells might look.^[^ ^63^ ^]^ This group adopted the name G FORCE PD after the meeting in New York in 2015.^[^ ^64^ ^]^ Over time, other groups have been invited on the principle that any academic group funded to pursue such an intervention would be welcome, although as one can imagine this has become more problematic of late as companies wishing to move into this therapeutic space have expanded (see Table 1). Nevertheless, this bringing together of like‐minded research groups with a common goal prepared to share data in an open way, has enabled the field to move forward with greater confidence and efficiency and we feel is an exemplar of how other novel therapies should be best discussed prior to commercial investment and the restrictions this brings with it. Perhaps, the most important impact of G FORCE PD has been the ability to openly discuss the preclinical approach we have each adopted within the regulatory framework of the country where the work is being done, as well as the proposed clinical trial – large elements of which have been informed by the TransEuro trial and the work leading up to that. This has also enabled us to facilitate discussions with regulators as to what constitutes the optimal preclinical package for their approval, the best clinical trial design, as well as advise commercial partners on what they should consider as they develop their own hPSC derived dopamine therapies. All of which has implications for any human stem cell therapy being considered for treating CNS diseases.

**TABLE 1:** Summary of trials planned or ongoing using hPSC derived dopamine cell replacements for PD.

Academic led trials			
	**Country of work**	**Type of cell**	**Current status of trial**
Stem‐PD	Sweden/UK	ESC	Started 2023, 6 patients grafted to date (*n* = 8 in trial)
CiRA trial	Japan	Allogeneic iPSC	Started 2018, last patient last visit Dec 2023, *n* = 7
**Academic led/compassionate single case study**			
Kwang Soo Kim/MGH team	USA	Autologous iPSC	*N* = 1, published.
**Company trials**			
**Company**	**Country of work**	**Type of cell**	**Current status of trial**
Aspen Neuroscience	USA	Autologous iPSC	First patient grafted in 2024
Bluerock Therapeutics/Bayer	USA	ESC	First trial of 12 patients completed in 2022
Novo Nordisk	Global	ESC	In set up
Oryon Cell Therapies	USA	Autologous iPSC	Filing for Phase I trial
Ryne Bio	USA	iPSC	In set up
S. Biomedics	South Korea	ESC	First trial of 12 patients completed in Feb 2024
Sumitomo	Japan now moving to the USA	iPSC	In set up in the USA

In this respect, one area that has emerged and which has proven to be a major issue is not the cell product itself but the device being used to deliver it to the brain. In the original hfVM trials, the tissue was grafted with a variety of devices that were typically borrowed from other neurosurgical procedures or made in‐house for such trials – for example, the Legradi‐Rehncrona (L‐R) device has been used since the 1980s in the successful trials with hfVM tissue in Lund. Although such an approach is fine when small academically led trials are being done on an exploratory basis, it is not a viable way forward especially when developing a therapy that could be used as a global standard of care if proven effective and safe. Thus, designing or deciding on the device for implanting the cells is a critical consideration, and in TransEuro, our failure to do this contributed in part to the negative outcome of the trial. In TransEuro we planned to use the L‐R device at both surgical sites (Cambridge and Lund), but Cambridge University Hospital would not allow us to do this as the L‐R device was not CE marked and thus could not be insured for use in the hospital. Therefore, an imitation device was made by the clinical engineering department in Cambridge based on the L‐R device. This device, although superficially similar, did not behave in the same way as the L‐R device, such that patients grafted in Cambridge had poorer graft survival and clinical outcomes compared to the patients grafted in Lund with the original L‐R device – presumably because delivery using the imitation device adversely affected the viability of implanted tissues. Thus, for all stem cell trials planned for PD, there is still a problem of delivery as no CE‐marked, approved device currently exists for this indication – and while the science linked to the generation of stem cell‐derived neurons of the right phenotype is clearly very exciting, the more practical and perhaps less scientifically exciting aspect of their delivery, is no less important. A point we will return to when we discuss our ongoing STEM PD trial.

## THE EMERGING CLINICAL TRIALS USING hPSC DERIVED DOPAMINE CELLS IN PwP 2018‐

Once robust patterning protocols for making hPSC derived midbrain DA neurons with high purity were established, developing the protocols for good manufacture practice (GMP) grade conditions suitable for early‐stage human trials were initiated,^[^ ^58^, ^59^, ^65^, ^66^ ^]^ along with methods for cryopreservation of the cell product.^[^ ^65^, ^67^ ^]^ Such cells have now been manufactured and extensively tested in vitro and in vivo for regulatory approval. To date, we are aware of a number of phase I/II clinical trials that have commenced (see Table 1). The first trial was initiated in Japan in 2018 and based on cells developed by Jun Takahashi and his team.^[^ ^68^ ^]^ This trial uses cells that are manufactured from one donor iPSC cell line for each surgery and involves cell sorting. The second trial was initiated in the US in 2021, conducted by BlueRock/Bayer using cells developed by the team led by Lorenz Studer and Viviane Tabar,^[^ ^59^ ^]^ and the third trial, STEM‐PD, was initiated by our teams at Lund University and the University of Cambridge in 2022, with the first surgery in Feb 2023 using cells developed by Malin Parmar and her team.^[^ ^18^ ^]^ These last two trials have both used batch‐produced, cryopreserved cells derived from hESCs. As all three trials are based on allogeneic cells, the trial participants are all receiving immune suppression for the first year post‐transplantation to prevent graft rejection. Other trials have started or are about to start (see Table 1) and a single case of an autologous IPSC derived dopamine cell transplant has been reported showing it was safe^[^ ^69^ ^]^.

Our own trial, STEM‐PD, is a single arm, first‐in‐human, phase I/IIa multicenter dose escalation trial to assess, at 12 months, the safety and tolerability of hESC‐derived DA progenitor cells grafted into the putamen of patients with moderate PD. Secondary endpoints include efficacy and imaging outcomes at 36 months. The cryopreserved STEM‐PD product has been quality tested for cell line identity, viability, yield, sterility, endotoxins, viruses, and unknown adventitious agents according to EU ATMP guidelines. The non‐clinical data for STEM‐PD includes safety studies in nude rats assessing toxicology, biodistribution, and tumorigenicity for up to 39 weeks post grafting as well as efficacy studies in 6‐OHDA lesioned nude rats. Moreover, the absence of 5HT neurons in the grafts has been confirmed. All non‐clinical data leading up to the regulatory approval in Europe under the EU Advanced Therapeutic Medicinal Product (ATMP) regulation and from the MHRA has been published.^[^ ^18^ ^]^

The patients in the STEM‐PD are recruited directly from the TransEuro observational natural history study which began in 2011. This means that the patients in the trial have been followed for extended periods of time with a well‐monitored disease course prior to this transplantation. The patients are being grafted with one of two doses of day 16 RC17 hES derived dopaminergic progenitors, using doses of cells calculated to give between 100 000 and 200 000 surviving midbrain dopaminergic neurons in the grafts. The cells are grafted in one surgical session with each patient having bilateral transplants placed into the posterior putamen, following which they are on standard whole organ immunosuppression for a year. The surgery is all being done in Lund with the L‐R device but with patients from Sweden and the UK. Follow‐up involves extensive clinical assessments, MRI, and dopamine PET imaging using protocols that are largely derived from the TransEuro study. To date, six patients have been transplanted, four at the lower dose of cells and two at the higher dose, with the expectation that the last of the eight patients will be grafted by October 2024.

## CONCLUSIONS AND THE FUTURE

**FIGURE 1:**
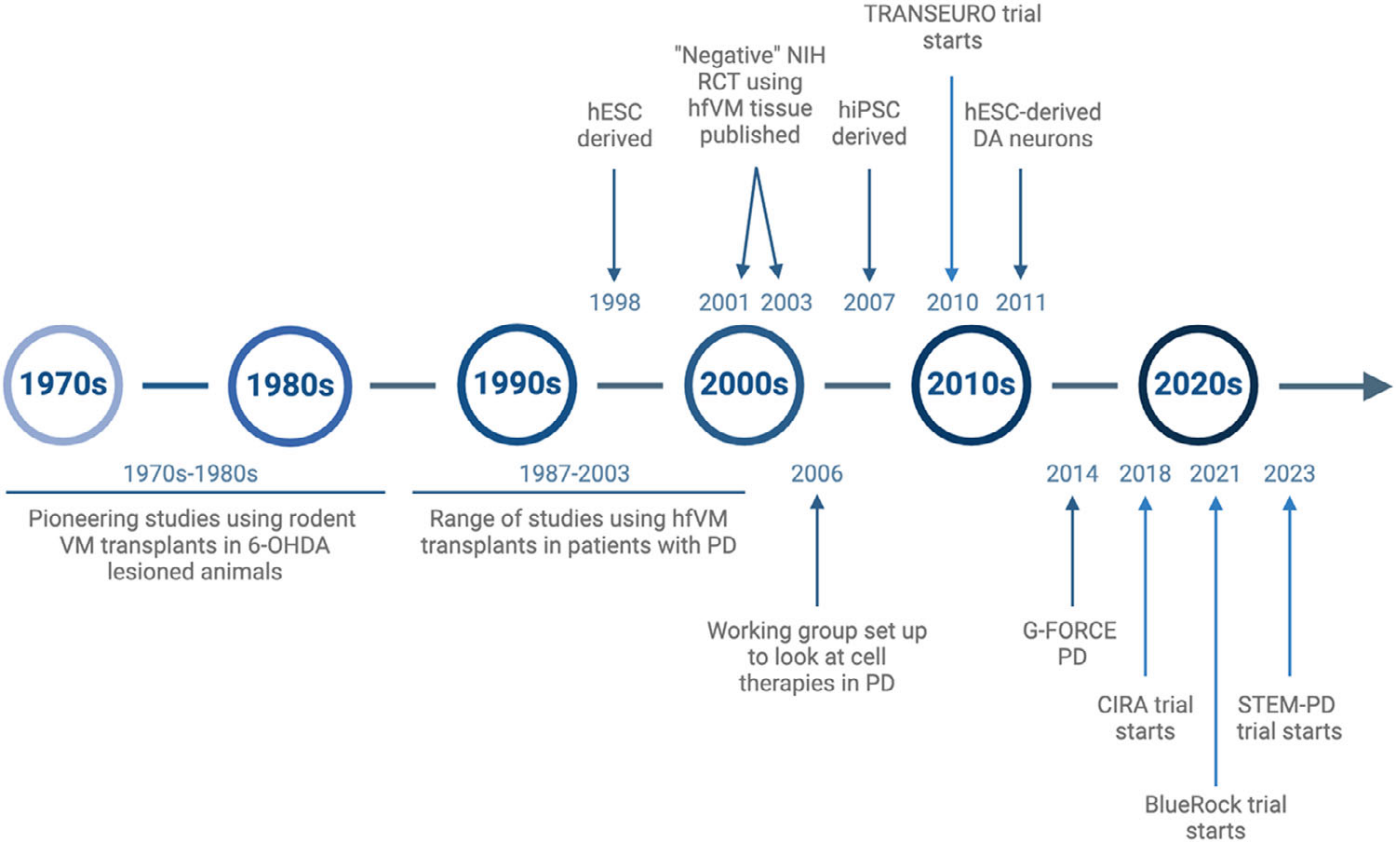
Brief history of dopamine cell grafting for Parkinson's disease.

In this article, we have given a history of the evolution of dopamine cell‐based therapies for PD which started almost exactly 50 years ago (see Figure 1). The original work was done before embryonic stem cells could be isolated and grown from mice (1981^[^ ^70^ ^]^) and then from humans in 1998,^[^ ^42^ ^]^ and was undertaken as a means to study the performance of fetal brain‐derived neuroblasts after transplantation to the brain, before expanding into proof of concept studies aimed at brain repair. These original rodent studies were groundbreaking, showing that neuroblasts derived from fetal tissue could be successfully implanted into the adult CNS where they could be shown to survive, differentiate normally, and appropriately innervate the host brain with functional effects. This led to the initiation of a number of trials adopting a similar approach in PwP which was again striking in what it showed – namely that grafting tissue from human fetal material through termination of pregnancies could be used to repair the aging adult PD brain through replacing the dopaminergic neurons lost within the nigrostriatal pathway. That such cells could survive, receive, and make connections with the host brain and exert major clinical benefits that lasted for many years or decades is a remarkable finding, although for this to translate into a therapy for clinical adoption, consistency, and scalability are needed – neither of which has been possible using the hfVM tissue source. Thus, for this approach to claim such a position, a more reliable source of cells was needed from which midbrain dopaminergic neurons could be made. This has become possible in recent years through our ability to derive and grow human ES and iPS cells coupled to a greater understanding of how to differentiate such cells into the nigral dopaminergic neuronal phenotype needed for use in PwP. As such we now sit at an important crossroads in the treatment of PD as these cells now enter clinical trials for the first time. An important factor is that, unlike fetal tissue transplants, this is being done with major pharma support which means that this approach can more quickly move to market if these early trials prove that the cells are safe and effective.

If this happens, where will we go next with dopamine cell therapies for PD? We would say that clinically this therapy may move towards first‐line treatment in subtypes of PwP, given in theory it would avoid patients having to take any anti‐PD drugs (at least for many years) and by so doing prevent PwP from developing drug‐related side effects, some of which are then treated with DBS. As such this therapy could transform the natural history of treated PD. In terms of the cell therapy itself, there is interest in making the cells resistant to the disease process through knocking out alpha synuclein^[^ ^71^, ^72^ ^]^ and/or making them less immunogenic by knocking out major immune epitopes such as HLA I and II (reviewed in Ref. [73]), or developing autologous therapies. Finally, there is great interest in using the technologies developed for turning hPSC into dopamine neurons to directly convert host glial cells into the dopaminergic neurons lost in PD. This direct in situ reprogramming is now being explored preclinically, albeit with limited efficacy and success to date,^[^ ^74^, ^75^ ^]^ but nevertheless offers great hope for the future, especially if these in situ generated cells are made disease resistant – as it would avoid many of the ethical and immunological issues that currently exist for hPSC based transplant therapies.

## CONFLICT OF INTEREST STATEMENT

The authors declare no conflicts of interest.
